# Supplementary figures and images for: Corallimorpharians are not “naked corals”: insights into relationships between Scleractinia and Corallimorpharia from phylogenomic analyses
Source: PeerJ. 2016 Oct 11;4:e2463. doi: 10.7717/peerj.2463 (PMC5068439; doi:10.7717/peerj.2463)

**Figure S1** Diagram showing the complete workflow from the data collection to analysis.


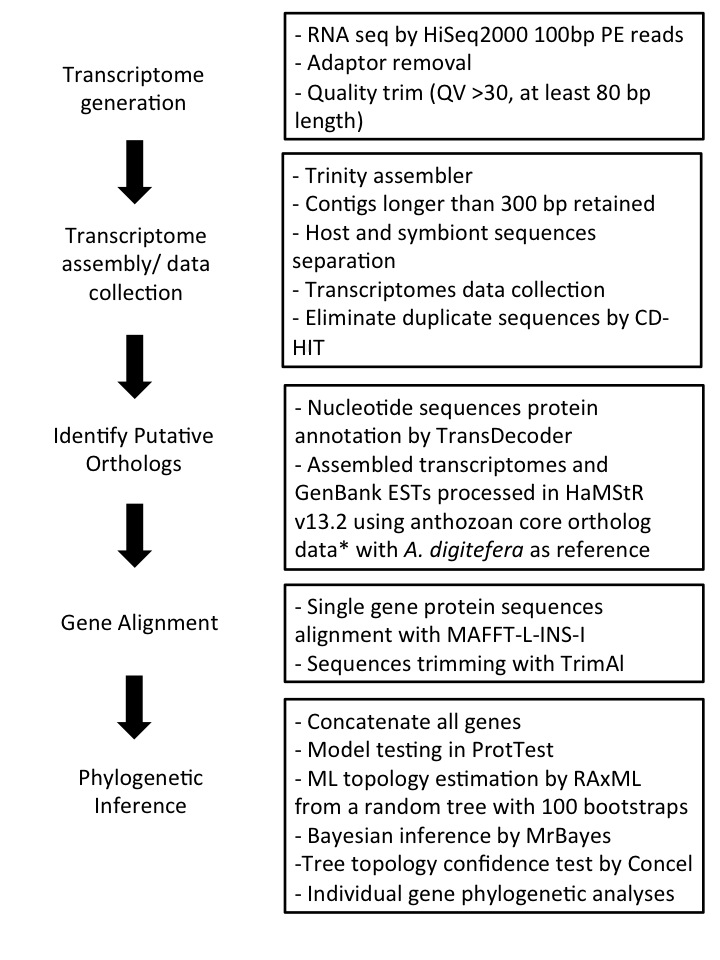

Supplement: Figure S1 — Diagram showing the complete workflow from the data collection to analysis. [file peerj-04-2463-s001.docx]
