# Supplementary material for: Corallimorpharians are not “naked corals”: insights into relationships between Scleractinia and Corallimorpharia from phylogenomic analyses
Source: PeerJ. 2016 Oct 11;4:e2463. doi: 10.7717/peerj.2463 (PMC5068439; doi:10.7717/peerj.2463)
Supplement: Figure S2 — ML tree obtained based on mitochondrial genes for a set of species similar to that used for the nuclear markers. The best-fit evolutionary models for aa mt matrix as JTT + G + F (lnL -36081.99) was used for the ML analyses with a discrete Gamma ( + G) distribution for 100 replicates. This analysis is consistent with the naked coral hypothesis, as in the case of previous analyses based on mitochondrial amino acid sequences. [file peerj-04-2463-s002.docx]

**Figure S2** ML tree obtained based on mitochondrial genes for a set of species similar to that used for the nuclear markers. The best-fit evolutionary models for aa mt matrix as JTT+G+F (lnL -36081.99) was used for the ML analyses with a discrete Gamma (+G) distribution for 100 replicates. This analysis is consistent with the naked coral hypothesis, as in the case of previous analyses based on mitochondrial amino acid sequences.

**
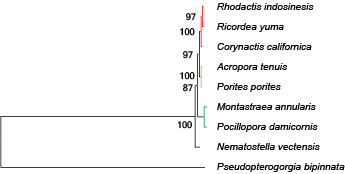
**
