# Supplementary material for: Corallimorpharians are not “naked corals”: insights into relationships between Scleractinia and Corallimorpharia from phylogenomic analyses
Source: PeerJ. 2016 Oct 11;4:e2463. doi: 10.7717/peerj.2463 (PMC5068439; doi:10.7717/peerj.2463)

**Figure S3** Amino acid (upper) and nucleotide (lower) composition of the nuclear dataset across the range of taxa studied. The values are averaged across the Complexa (n=3), Robusta (n=6), Corallimorpharia (n=3), Actiniaria (n=2) and Octocorallia (n=1) and are relatively homogenous across the lineages.


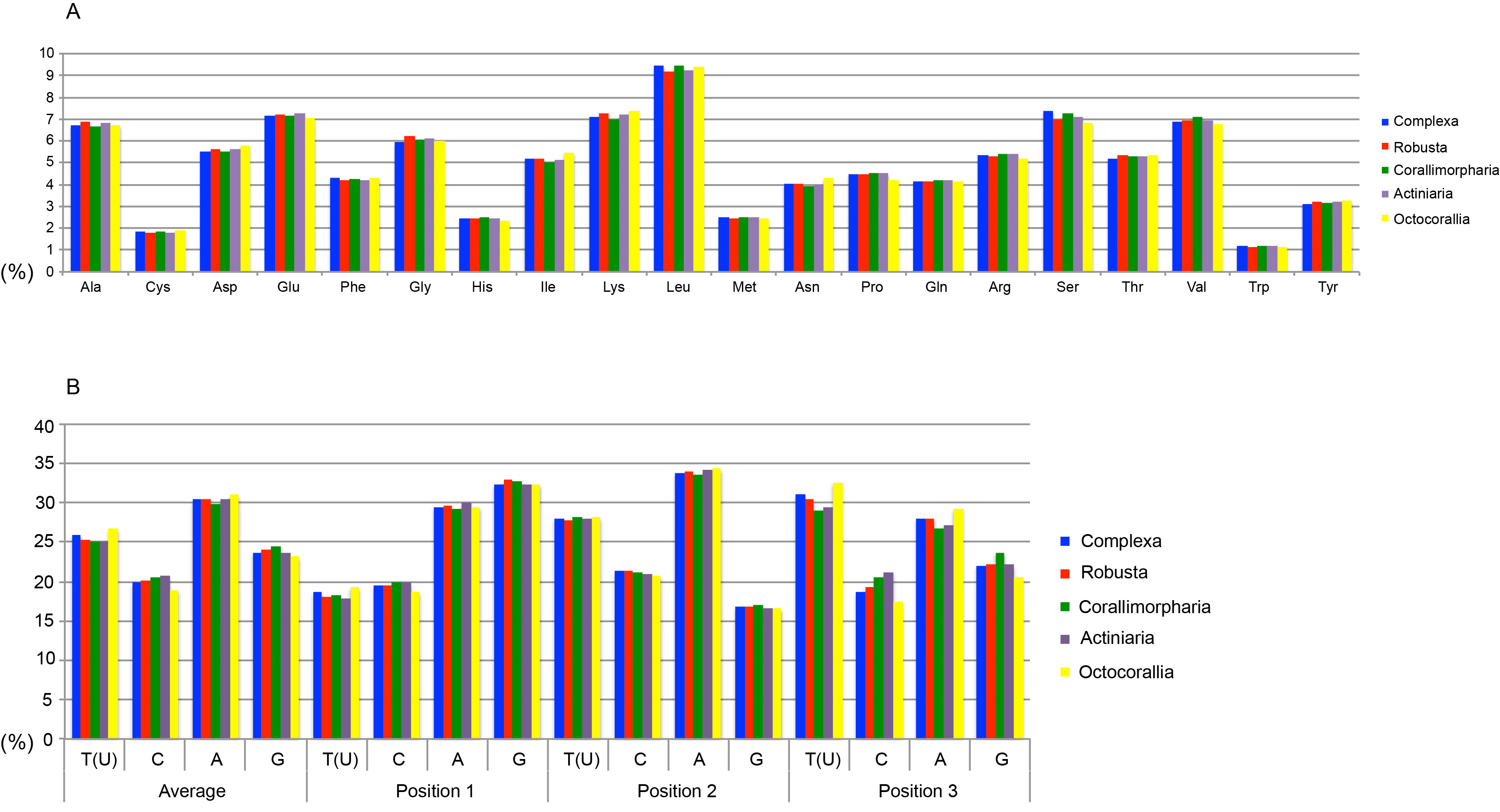

Supplement: Figure S3 — Amino acid (upper) and nucleotide (lower) composition of the nuclear dataset across the range of taxa studied. The values are averaged across the Complexa (n=3), Robusta (n=6), Corallimorpharia (n=3), Actiniaria (n=2) and Octocorallia (n=1) and are relatively homogenous across the lineages. [file peerj-04-2463-s003.docx]
