# Supplementary material for: Corallimorpharians are not “naked corals”: insights into relationships between Scleractinia and Corallimorpharia from phylogenomic analyses
Source: PeerJ. 2016 Oct 11;4:e2463. doi: 10.7717/peerj.2463 (PMC5068439; doi:10.7717/peerj.2463)
Supplement: Figure S4 — Primary concordance tree of the 291 nuclear genes from 15 anthozoans. Numbers on edges are posterior mean concordance factors and their 95% credibility intervals. The numbers above and below the edges are the sample-wide concordance factors, and genome-wide concordance factors respectively. [file peerj-04-2463-s004.docx]

**Figure S4** Primary concordance tree of the 291 nuclear genes from 15 anthozoans. Numbers on edges are posterior mean concordance factors and their 95% credibility intervals. The numbers above and below the edges are the sample-wide concordance factors, and genome-wide concordance factors respectively.
