# Supplementary material for: Corallimorpharians are not “naked corals”: insights into relationships between Scleractinia and Corallimorpharia from phylogenomic analyses
Source: PeerJ. 2016 Oct 11;4:e2463. doi: 10.7717/peerj.2463 (PMC5068439; doi:10.7717/peerj.2463)
Supplement: Table S1 [file peerj-04-2463-s005.docx]

**Table S1** Data sources

|  | **Family** | **Species** | **Source** | **Data type** | **# ortholog** |
| --- | --- | --- | --- | --- | --- |
| **Complex** | Acroporidea | *Acropora millepora* | Moya et al, 2012^1^ | Transcriptome | 1609 |
|  | Acroporidea | *Acropora digitifera* | Shinzato et al. 2011^2^ | Genome | Primer/Reference |
|  | Poritidea | *Porites australiensis* | Shinzato et al. 2014^3^ | Transcriptome | 1296 |
| **Robust** | Fungiidae | *Fungia scutaria* | Meyer and Weis lab^4^ | Transcriptome | 1511 |
|  | Pocilloporidae | *Madracis auretenra* | Meyer and Weis lab^4^ | Transcriptome | 1442 |
|  | Montastraeidae | *Montastraea cavernosa* | Meyer and Weis lab^4^ | Transcriptome | 1505 |
|  | Pocilloporidae | *Pocillopora damicornis* | Traylor-Knowles et al. 2011^5^ | Transcriptome | 1203 |
|  | Merulinidae | *Platygyra carnosus* | Sun et al. 2013^6^ | Transcriptome | 1561 |
|  | Favinae | *Pseudodiploria strigosa* | Meyer and Weis lab^4^ | Transcriptome | 1358 |
| **Corallimorph** | Corallimorphidae | *Corynactis australis* | Lin et al. submitted. | Transcriptome | 1481 |
|  | Discosomatidae | *Rhodactis indosinesis* | Lin et al. submitted. | Transcriptome | 1261 |
|  | Ricordeidae | *Ricordea yuma* | Lin et al. submitted. | Transcriptome | 1401 |
| **Actiniaria** |  | *Nematostella vectensis* | NCBI PRJNA19965 | Genome | Primer |
|  |  | *Anthopleura elegantissima* | Meyer and Weis lab^4^ | Transcriptome | 1448 |
| **Octocorallia** |  | *Gorgonia ventalina* | Colleen et al. 2013^7^ | Transcriptome | 1421 |
| **Hydra** |  | *Hydra magnipapillata* |  | Genome | Primer |

1. Moya A, Huisman L, Ball EE, Hayward DC, Grasso LC, Chua CM, Woo HN, Gattuso JP, Forêt S, Miller DJ. Whole transcriptome analysis of the coral Acropora millepora reveals complex responses to CO₂-driven acidification during the initiation of calcification. Mol Ecol. 2012 May;21(10):2440-54.
2. Chuya Shinzato, Eiichi Shoguchi, Takeshi Kawashima, Mayuko Hamada, Kanako Hisata, Makiko Tanaka, Manabu Fujie, Mayuki Fujiwara, Ryo Koyanagi, Tetsuro Ikuta, Asao Fujiyama, David J. Miller, Nori Satoh. 2011. Using the Acropora digitifera genome to understand coral responses to environmental change. *Nature* 476: 320–323.
3. Chuya Shinzato, Mayuri Inoue, Makoto Kusakabe. A Snapshot of a Coral “Holobiont”: A Transcriptome Assembly of the Scleractinian Coral, Porites, Captures a Wide Variety of Genes from Both the Host and Symbiotic Zooxanthellae. PLoS ONE 9: e85182.
4. Meyer and Weis lab: <http://people.oregonstate.edu/~meyere/data.html>
5. Nikki Traylor-Knowles, Brian R. Granger, Tristan J. Lubinski, Jignesh R. Parikh, Sara Garamszegi, Yu Xia, Jarrod A. Marto, Les Kaufman, John R Finnerty. 2011. Production of a reference transcriptome and transcriptomic database (PocilloporaBase) for the cauliflower coral, *Pocillopora damicornis*. *BMC Genomics* 12: 585.
6. Sun J, Chen Q, Lun JCY, Xu J, Qiu J-W. 2013. PcarnBase: development of a transcriptomic database for the brain coral *Platygyra carnosus*. *Mar Biotechnol.* 15:244-251.
7. Colleen A. Burge, Morgan E. Mouchka, C. Drew Harvell, Steven Roberts. 2013. Immune response of the Caribbean sea fan, *Gorgonia ventalina*, exposed to an *Aplanochytrium* parasite as revealed by transcriptome sequencing. *Front Physiol.* 4: 180.
