# Supplementary material for: Corallimorpharians are not “naked corals”: insights into relationships between Scleractinia and Corallimorpharia from phylogenomic analyses
Source: PeerJ. 2016 Oct 11;4:e2463. doi: 10.7717/peerj.2463 (PMC5068439; doi:10.7717/peerj.2463)
Supplement: Table S2 [file peerj-04-2463-s006.docx]

**Table S2** Transcriptome assembly summary

|  | Number of contigs | N50 | Mean | Max |
| --- | --- | --- | --- | --- |
| *Corynactis australis* | 74,176 | 1,384 | 905 | 21,094 |
| *Rhodactis indosinesis* | 69,648 | 1,945 | 1,055 | 27,136 |
| *Ricordea yuma* | 50,070 | 1,786 | 1,066 | 13,850 |
