# Supplementary material for: Corallimorpharians are not “naked corals”: insights into relationships between Scleractinia and Corallimorpharia from phylogenomic analyses
Source: PeerJ. 2016 Oct 11;4:e2463. doi: 10.7717/peerj.2463 (PMC5068439; doi:10.7717/peerj.2463)
Supplement: Table S3 — Nucleotide substitution saturation tests on the 291 nuclear protein-coding genes.The nucleotide substitution saturation test calculates an index of substitution saturation (Iss), which is compared to a critical value computed for a symmetrical (Iss.cSym) or extremely asymmetrical (Iss.c asym) tree topology. Pinv: proportion of invariant sites. Iss: index of substitution saturation. Iss.c sym: critical value for symmetrical tree topology. Iss.c asym: critical value for extremely asymmetric tree topology. T: T value. DF: degrees of freedom. P: probability that Iss is significantly different from the critical value (Iss.c sym or Iss.c asym). Two-tailed tests were used. [file peerj-04-2463-s007.docx]

**Table S3** Nucleotide substitution saturation test on the 291 nuclear protein-coding genes. The nucleotide substitution saturation test calculates an index of substitution saturation (Iss), which is compared to a critical value computed for a symmetrical (Iss.cSym) or extremely asymmetrical (Iss.c asym) tree topology. Pinv: proportion of invariant sites. Iss: index of substitution saturation. Iss.c sym: critical value for symmetrical tree topology. Iss.c asym: critical value for extremely asymmetric tree topology. T: T value. DF: degrees of freedom. P: probability that Iss is significantly different from the critical value (Iss.c sym or Iss.c asym). Two-tailed tests were used.
