# Supplementary material for: Corallimorpharians are not “naked corals”: insights into relationships between Scleractinia and Corallimorpharia from phylogenomic analyses
Source: PeerJ. 2016 Oct 11;4:e2463. doi: 10.7717/peerj.2463 (PMC5068439; doi:10.7717/peerj.2463)
Supplement: Table S4 — Compositional biases of the 291 nuclear genes of the anthozoan Orders included in the present study. Compositional biases of the mitochondrial genes are given in parentheses and are based on Kitahara et al. (2014). The mitochondrial compositions differ markedly in Robusta compared to other Hexacorallia, whilst the nuclear composition is homogeneous across the Anthozoa. [file peerj-04-2463-s008.docx]

**Table S4** Compositional biases of the 291 nuclear genes of the anthozoan Orders included in the present study. Compositional biases of the mitochondrial genes are given in parentheses and are based on Kitahara et al. (2014). The mitochondrial compositions differ markedly in Robusta compared to other Hexacorallia, whilst the nuclear composition is homogeneous across the Anthozoa.

|  |  |  | **Nucleotide** | | | **Protein** | | |
| --- | --- | --- | --- | --- | --- | --- | --- | --- |
| **Sub-class** | **Order** | **Group** | G+C(%) | A+T(%) |  | FYMINK(%) | GARP(%) | FYMINK/ GARP |
| Octocorallia | Alcyonacea |  | 42.20 | 57.80 |  | 27.16 | 22.13 | 1.227 |
|  |  |  | (35.33) | (64.66) |  | (30.54) | (21.96) | (1.39) |
| Hexacorallia | Actiniaria |  | 44.43 | 55.57 |  | 26.15 | 22.86 | 1.144 |
|  |  |  | (37.95) | (62.05) |  | (29.92) | (22.47) | (1.33) |
|  | Corallimorpharia |  | 45.06 | 54.94 |  | 25.83 | 22.69 | 1.138 |
|  |  |  | (37.95) | (62.05) |  | (28.73) | (22.15) | (1.29) |
|  | Scleractinia | Complexa | 43.60 | 56.40 |  | 26.27 | 22.46 | 1.170 |
|  |  |  | (37.59) | (62.41) |  | (29.42) | (21.81) | (1.34) |
|  |  | Robusta | 44.12 | 55.88 |  | 26.33 | 22.82 | 1.154 |
|  |  |  | (31.2) | (68.8) |  | (33.71) | (19.36) | (1.74) |
